# Comparison of the Risk of Gastrointestinal Bleeding among Different Statin Exposures with Concomitant Administration of Warfarin: Electronic Health Record-Based Retrospective Cohort Study

**DOI:** 10.1371/journal.pone.0158130

**Published:** 2016-07-07

**Authors:** Dahye Shin, Dukyong Yoon, Sun Gyo Lim, Ji Man Hong, Rae Woong Park, Jin Soo Lee

**Affiliations:** 1 Department of Biomedical Informatics, Ajou University School of Medicine, Suwon, Gyeonggi-do, Republic of Korea; 2 Department of Gastroenterology, Ajou University School of Medicine, Suwon, Gyeonggi-do, Republic of Korea; 3 Department of Neurology, Ajou University School of Medicine, Suwon, Gyeonggi-do, Republic of Korea; 4 Department of Biomedical Sciences, Ajou University Graduate School of Medicine, Suwon, Gyeonggi-do, Republic of Korea; University of Manitoba, CANADA

## Abstract

**Background and Objective:**

Patients who should be treated with both warfarin and a statin are frequently seen in vascular clinics. The risk for bleeding and potential drug interactions should be considered when prescribing both medications together. This study aimed to compare the risk for gastrointestinal bleeding among different statin exposures with concomitant administration of warfarin.

**Materials and Methods:**

This is a single-hospital retrospective cohort study. We included patients who were concomitantly exposed to one of four statins (pravastatin, simvastatin, atorvastatin, and rosuvastatin) and warfarin for up to 2 years (730 days). The observation period ended when a gastrointestinal bleeding event occurred or the observation was censored. Within-class comparisons were used, and 1:1 matching using a propensity score was performed for comparisons between each statin and all of the other statins. Kaplan-Meier analyses with log-rank tests and Cox proportional hazard regression analyses were conducted to determine associations with the risk of gastrointestinal bleeding.

**Results:**

Data were analyzed for 1,686 patients who were concomitantly administered a statin and warfarin. Log-rank tests for the gastrointestinal bleeding-free survival rate showed that the risk for gastrointestinal bleeding was significantly lower in the pravastatin group (*p* = 0.0499) and higher in the rosuvastatin group (*p* = 0.009). In the Cox proportional hazard regression analysis, the hazard ratio of 5.394 for gastrointestinal bleeding based on statin exposure in the rosuvastatin group was significant (95% confidence interval, 1.168–24.916).

**Conclusions:**

There was a relatively high risk of gastrointestinal bleeding with rosuvastatin when administered concomitantly with warfarin.

## Introduction

Warfarin is used to prevent embolic events in the vascular system that can cause an ischemic stroke, peripheral arterial occlusion, deep vein thrombosis, or pulmonary embolism [1–4]. Although new oral anticoagulants have recently been developed, they are only available for patients with atrial fibrillation or deep vein thromboses [5]. Therefore, warfarin is still widely indicated.

Statins are used to prevent progression of atherosclerosis in the arterial system [6, 7] and are particularly important for patients with coronary arterial disease or atherosclerotic ischemic stroke [8–11]. In addition, statins are used for primary prevention for patients with high cholesterol levels.

Patients who should be treated with both warfarin and a statin are frequently seen in vascular clinics. When prescribing both medications together, the bleeding risk and a potential drug interaction should be considered. Gastrointestinal (GI) bleeding is one of the most frequent complications of warfarin [12] and can occur when the warfarin level exceeds the target range [13–15]; the warfarin level can fluctuate owing to dietary factors, other medications, or some genetic factors [16]. Co-administration of some statins reportedly increases the risk of GI bleeding, as measured during a 1-month of co-administration of warfarin and statins [17]. However, there is controversy regarding this side effect, because other evidence suggests that statins might decrease GI bleeding in patients treated with warfarin [18, 19].

Electronic health records (EHRs) are expected to be a useful source of data for epidemiologic studies because they contain detailed information on clinical events and related medications [20–22]. The quantity of data in EHRs is continuously increasing with additions from daily clinical practice [23]. Therefore, EHRs can provide greater accessibility, accuracy, and completeness of clinical information to researchers. Using EHR data, this study aimed to compare the risk of GI bleeding among four different statins (simvastatin, atorvastatin, pravastatin, and rosuvastatin) when co-administered with warfarin for at least a 30-day period and adjusting for other concomitant medications and baseline characteristics.

## Materials and Methods

### Data source

Data from the EHR database for a 1,096-bed Korean tertiary teaching hospital were used, including basic patient information, prescriptions, and laboratory test results collected between January 1996 and August 2013; these data included 116,611,087 prescriptions and 158,122,485 laboratory test results for 1,272,977 patients. This study was reviewed and approved by the local Institutional Review Board (Ajou Institutional Review Board [MED-MDB-13-101]). Patient information was anonymized and de-identified prior to analysis.

### Patient selection and cohort definition

**Fig 1:**
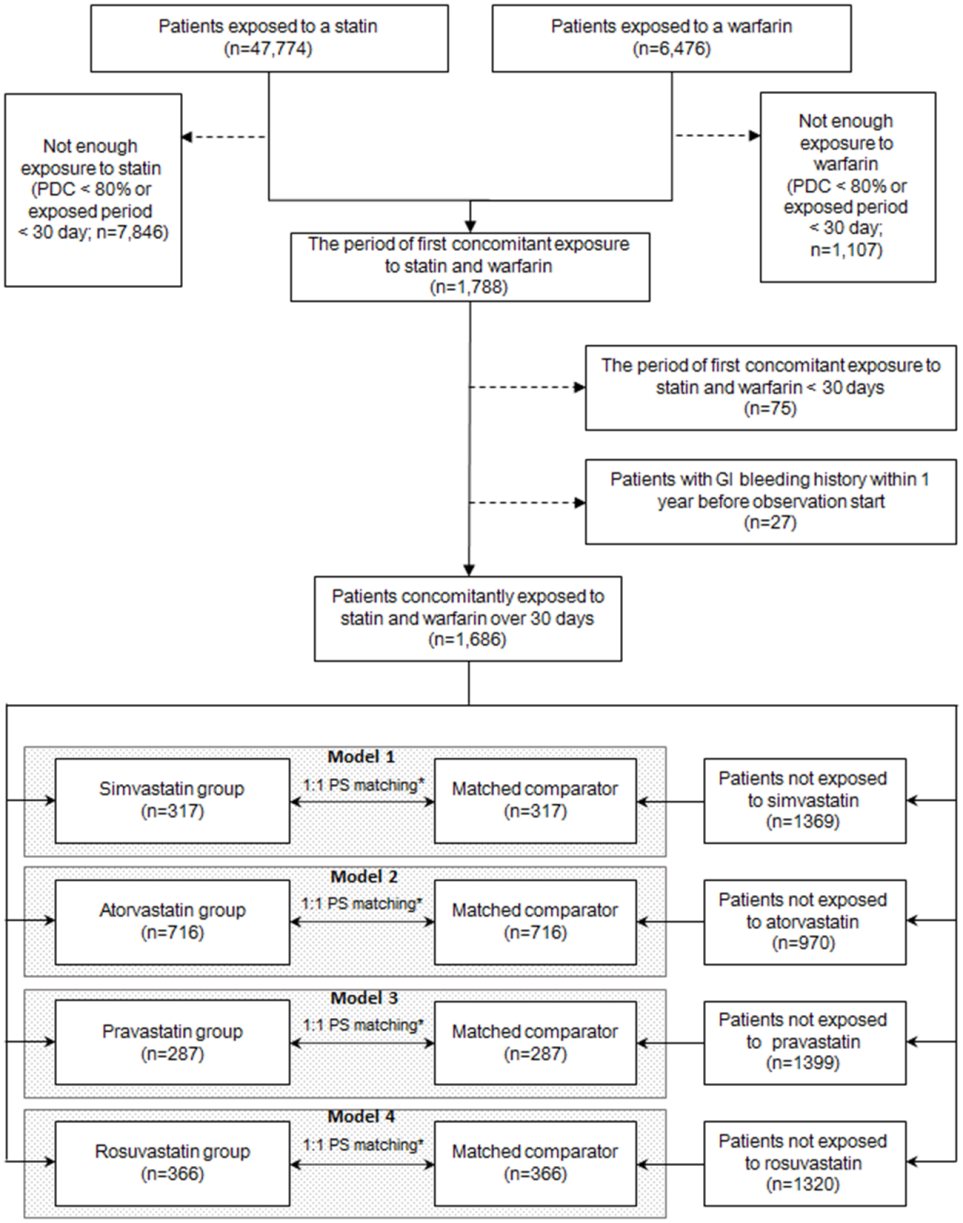
Overview of the study design to compare the gastrointestinal bleeding risk between 1 of 4 statins concomitantly administered with warfarin. All patients who were administered warfarin or a statin between January 1996 and August 2013 in a Korean university hospital were enrolled. Patients were divided into four groups based on the prescribed statin. Within-class comparisons were conducted after 1:1 propensity score (PS) matching. *The propensity scores were calculated using age; sex; Charlson comorbidity index; antithrombotic, nonsteroidal anti-inflammatory drug, steroid, or fibrate use for >30 days during the observation period; underlying liver cirrhosis; and underlying coagulation disorders. GI, gastrointestinal; PDC, proportion of days covered.

This is a single-hospital retrospective cohort study. Data were extracted for patients who were concomitantly administered one of the four target statins (simvastatin, atorvastatin, pravastatin, or rosuvastatin) and warfarin (Fig 1). Among the patients who were exposed to statins and warfarin, we included patients for whom each prescription was administered for over 30 days of the first co-administration period and the proportion of days covered, calculated as the prescription supply (days)/duration of continuous administration, was >80%. Patients were excluded for the following reasons: lack of continuous statin administration lasting ≥30 days or history of GI bleeding during the year prior to the first co-administration.

**Fig 2:**
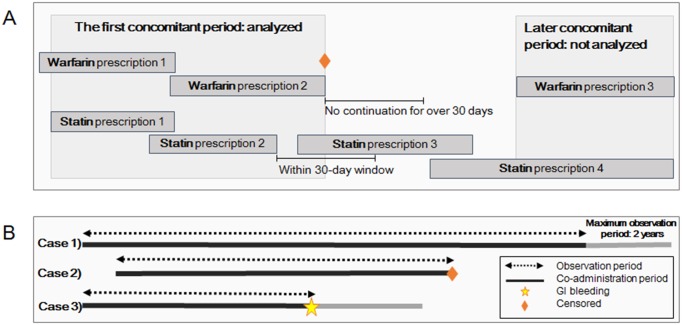
Definition of continuous administration, co-administration, and observation period for statins and warfarin. (A) Continuous administration was defined as repeated prescriptions of the same medication with a <30-day interval between the end of the previous prescription and start of the following prescription. The co-administration period was defined as overlapping continuous administration of both a statin and warfarin. (B) The observation period lasted up to 2 years from the start of the statin and warfarin co-administration and ended when a gastrointestinal (GI) bleeding event occurred or observation was censored.

The included patients were divided into four groups based on the statin type. The observation period (maximum, 2 years) was defined as the period that included continuous administration of both warfarin and statin and started on the first day of co-administration (Fig 2). The observation period ceased when medication was continued for >30 days and if GI bleeding occurred. Only the first co-administration period was included (incident user design). We did not include periods after switching from one statin to another or a >30-day lapse in administration of any drug.

GI bleeding was identified in 3 steps. First, records were searched for corresponding International Classification of Diseases, 10th Revision codes, such as bleeding in the esophagus, stomach, or duodenum (subdivisions .0–.6 of K25–K28, K29.0, K62.5, K92.0–2, and I85.0). Second, GI bleeding was identified using institutional codes for positive stool occult blood. Third, GI bleeding was identified using other institutional codes such as control of endoscopic bleeding in the upper or lower GI tract.

The following information was collected for the observation period: demographic characteristics (age and sex); age-adjusted Charlson Comorbidity Index (CCI); proportion of time spent in the therapeutic range (international normalized ratio [INR] 2–3), as calculated using the Rosendaal method [24]; and >30-day exposure to other antiplatelets, non-steroidal anti-inflammatory drugs (NSAIDs), steroids, or fibrate. The age-adjusted CCI is a weighted index of comorbidity based on age and the presence of diverse medical conditions including myocardial infarction and cerebrovascular disease. Diagnostic codes for liver cirrhosis (K70-K77) or coagulation disorders (D65-D69) before the observation period were collected. To compare serologic test results among the statin groups, the following information was also collected from 90 days before the observation period to the start of the observation period, as the baseline values, and during the observation period, as the follow-up values: total cholesterol, high-density lipoprotein, low-density lipoprotein, triglyceride, aspartate transaminase, alanine transaminase, and creatine kinase levels.

### Statistical analysis

Categorical and continuous variables were compared among the groups using Chi-squared tests and analysis of variance (ANOVA), respectively. To evaluate the risk of co-administration of different statins and warfarin on GI bleeding, within-class comparisons were conducted; the data for each statin group were compared with combined data for the other groups (Fig 1). To reduce the selection bias in these comparisons, we performed 1:1 matching using a propensity score that was derived from age; sex; CCI; antiplatelet, NSAID, steroid, or fibrate use for >30 days during the observation period; underlying liver cirrhosis; and underlying coagulation disorders. Kaplan-Meier analyses with log-rank tests for GI bleeding-free survival rate and Cox proportional hazard regression analyses were also conducted. The Cox proportional hazard regression analyses were adjusted for age, sex, CCI, proportion of time spent within the therapeutic range, temporal order of warfarin and statin prescription (e.g., warfarin prescription prior to statin prescription), and use of antiplatelets, NSAIDs, or steroids for >30 days during the observation period. Statistical significance was defined as *p* < 0.05. This study is reported following the STROBE statement (S1 File).

### Software tools

We used Eclipse 3.7.1 tools (IBM, Riverton, NJ) for JAVA programming and MS-SQL 2010 (Microsoft, Redmond, WA) as the database management system. The R package (R Development Core Team, Vienna, Austria) and PASW Statistic 18 (SPSS Inc., Chicago, IL) were used for statistical analyses.

## Results

### Characteristics of the overall population

We identified 1,686 patients who were concomitantly administered a statin with warfarin for ≥30 days, resulting in 287 patients who were administered pravastatin, 317 patients who were administered simvastatin, 716 patients who were administered atorvastatin, and 366 patients who were administered rosuvastatin (Table 1). Before matching, patients in the atorvastatin group were slightly older and had a higher CCI, and concomitant antiplatelets were prescribed more frequently in the simvastatin and rosuvastatin groups. The baseline serologic test results did not differ among the groups, except for triglyceride levels, which was higher in the simvastatin group.

**Table 1:** Baseline characteristics and laboratory results at baseline and during the observation period for the four statin groups.

Variables	Pravastatin	Simvastatin	Atorvastatin	Rosuvastatin	*p*-value
**Total number (n)**	287	317	716	366	-
**Age (years)**	61.23 ± 13.74	61.03 ± 12.09	63.32 ± 13.22	62.29 ± 13.85	0.031^§^
**Men**	176 (61.30%)	187 (59.00%)	424 (59.20%)	234 (63.90%)	0.443^†^
**Nonsteroidal anti-inflammatory drugs**	25 (8.70%)	19 (6.00%)	56 (7.80%)	21 (5.70%)	0.353^†^
**Fibrates**	6 (2.10%)	9 (2.80%)	18 (2.50%)	4 (1.10%)	0.386^†^
**Steroids**	12 (4.20%)	11 (3.50%)	19 (2.70%)	15 (4.10%)	0.511^†^
**Antiplatelets**	141 (49.10%)	206 (65.00%)	370 (51.70%)	226 (61.70%)	0.000^†^
**Liver cirrhosis history**	10 (3.50%)	16 (5.00%)	34 (4.70%)	21 (5.70%)	0.607^†^
**Coagulopathy history**	2 (0.70%)	13 (4.10%)	16 (2.20%)	9 (2.50%)	0.054^†^
**Gastrointestinal bleeding history**	4 (1.4%)	7 (2.2%)	16 (2.2%)	11 (3.0%)	0.592^†^
**Charlson comorbidity index**	3.97 ± 1.923	3.59 ± 1.803	4.11 ± 1.913	3.91 ± 1.954	0.001^§^
**Proportion of time in the therapeutic range of warfarin**	0.45 ± 0.337 (n = 242)	0.42 ± 0.349 (n = 252)	0.43 ± 0.336 (n = 632)	0.47 ± 0.339 (n = 325)	0.217^§^
**Baseline**					
**Total cholesterol level (mg/dL)**	186.03 ± 56.389 (n = 243)	194.61 ± 50.690 (n = 219)	176.20 ± 46.883 (n = 601)	177.98 ± 50.969 (n = 298)	0.000^§^
**HDL cholesterol level (mg/dL)**	45.59 ± 12.025 (n = 216)	45.36 ± 11.01 (n = 180)	45.95 ± 12.700 (n = 540)	45.04 ± 13.820 (n = 266)	0.806^§^
**LDL cholesterol level (mg/dL)**	111.59 ± 38.412 (n = 213)	116.39 ± 40.408 (n = 167)	104.96 ± 36.949 (n = 512)	109.56 ± 40.502 (n = 253)	0.005^§^
**Triglyceride level (mg/dL)**	144.28 ± 123.579 (n = 221)	167.24 ± 125.948 (n = 181)	140.19 ± 114.487 (n = 540)	131.34 ± 96.051 (n = 266)	0.011^§^
**AST level (U/L)**	32.86 ± 31.059 (n = 240)	31.90 ± 20.873 (n = 212)	33.65 ± 35.137 (n = 598)	34.50 ± 34.968 (n = 293)	0.829^§^
**ALT level (U/L)**	35.50 ± 31.029 (n = 240)	30.75 ± 18.394 (n = 212)	31.19 ± 31.243 (n = 597)	30.69 ± 37.551 (n = 293)	0.236^§^
**CK level (U/L)**	156.93 ± 250.904 (n = 189)	190.00 ± 297.481 (n = 122)	228.86 ± 970.817 (n = 467)	346.74 ± 1784.855 (n = 218)	0.314^§^
**During the observation period**					
**Total cholesterol level (mg/dL)**	166.60 ± 44.162 (n = 148)	155.08 ± 36.376 (n = 184)	144.89 ± 34.970 (n = 459)	139.38 ± 36.628 (n = 239)	0.000^§^
**HDL cholesterol level (mg/dL)**	47.33 ± 12.838 (n = 118)	47.82 ± 11.799 (n = 142)	46.12 ± 11.662 (n = 351)	47.94 ± 13.984 (n = 188)	0.322^§^
**LDL cholesterol level (mg/dL)**	93.44 ± 35.210 (n = 111)	82.70 ± 30.595 (n = 123)	75.80 ± 27.190 (n = 331)	71.01 ± 28.007 (n = 176)	0.000^§^
**Triglyceride level (mg/dL)**	166.96 ± 101.023 (n = 120)	157.35 ± 85.476 (n = 151)	133.84 ± 84.371 (n = 357)	131.35 ± 79.315 (n = 190)	0.000^§^
**AST level (U/L)**	30.83 ± 25.290 (n = 139)	32.21 ± 24.175 (n = 177)	31.67 ± 29.686 (n = 460)	33.85 ± 21.239 (n = 264)	0.657^§^
**ALT level (U/L)**	32.04 ± 39.073 (n = 139)	31.42 ± 23.799 (n = 177)	31.05 ± 37.966 (n = 460)	35.03 ± 27.492 (n = 264)	0.474^§^
**CK level (U/L)**	306.15 ± 1054.835 (n = 56)	164.53 ± 272.454 (n = 73)	142.08 ± 239.370 (n = 257)	166.21 ± 386.900 (n = 144)	0.096^§^

HDL, high-density lipoprotein; LDL, low-density lipoprotein; AST, aspartate transaminase; ALT, alanine transaminase; CK, creatine kinase

Values are presented as n (%) or mean ± standard deviation. *P* values were determined using ANOVA^§^ or Chi-squared tests.^†^

During the observation period, the proportion of time spent within the therapeutic range of warfarin did not differ among the groups: pravastatin, 0.45 ± 0.34; simvastatin, 0.42 ± 0.35; atorvastatin, 0.43 ± 0.34; and rosuvastatin, 0.47 ± 0.34 (*p* = 0.217). Mean total cholesterol levels during the observation period were 167 ± 44 (rosuvastatin), 155 ± 36 (atorvastatin), 145 ± 35 (simvastatin), and 139 ± 37 mg/dL (pravastatin) (*p* < 0.001). Low-density cholesterol (*p* < 0.001) and triglyceride (*p* < 0.001) levels showed a similar pattern as that of total cholesterol levels. There was no significant difference in high-density cholesterol levels among the groups (*p* = 0.322).

### GI bleeding risks in a 1:1 matched population

**Fig 3:**
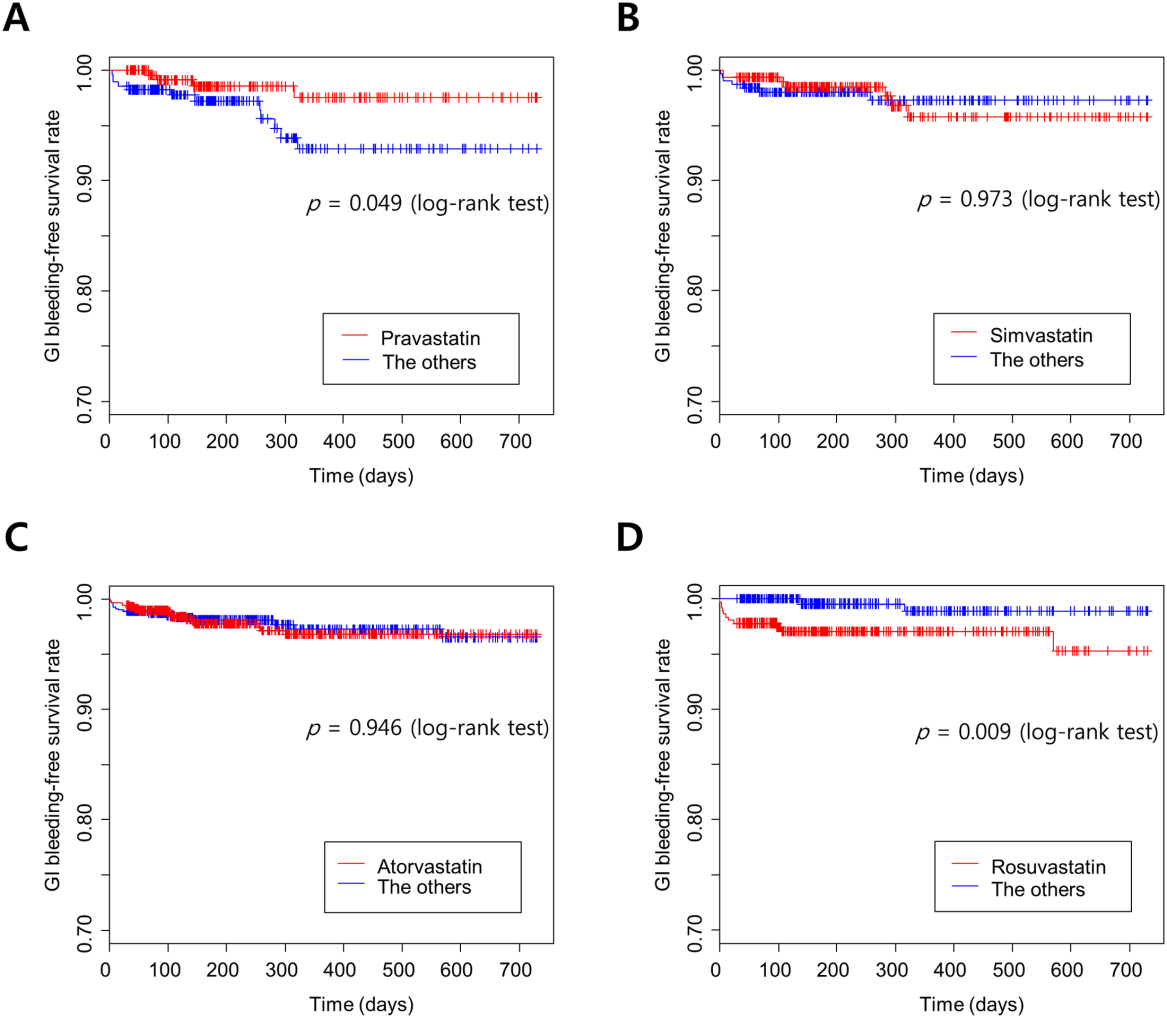
Kaplan Meier curves for the gastrointestinal bleeding-free survival rate according to exposure to four different statins concomitantly administered with warfarin. (A) Pravastatin vs. the other statins, (B) Simvastatin vs. the other statins, (C) Atorvastatin vs. the other statins, (D) Rosuvastatin vs. the other statins.

The incidence of GI bleeding is listed in the Table 2. After matching each group with a comparator, the Kaplan Meier curves for GI bleeding-free survival rate and log-rank tests resulted in a significantly lower risk of GI bleeding in the pravastatin group (*p* = 0.0499) and a significantly higher risk of GI bleeding in the rosuvastatin group (*p* = 0.009) (Fig 3). At the time of the GI bleeding event, the mean doses of pravastatin, simvastatin, atorvastatin, and rosuvastatin were 20 mg, 23 mg, 14 mg, and 17 mg, respectively.

**Table 2:** Incidence of gastrointestinal bleeding in patients administered both warfarin and a statin, based on the 3 steps of identification.

	1. ICD-10 codes for location of bleeding				2. Codes for stool blood	3. Codes for control of endoscopic bleeding	
	Upper	Lower	Upper/ lower	Variceal	Positive occult blood test	Upper gastrointestinal tract	Lower gastrointestinal tract
**Pravastatin**	2	0	1	0	0	1	0
**Simvastatin**	2	1	1	0	3	0	0
**Atorvastatin**	5	2	0	0	7	2	0
**Rosuvastatin**	2	2	0	0	5	2	0

ICD, International Classification of Diseases

In the Cox proportional hazard regression analysis for GI bleeding (Table 3), the hazard ratio (HR) for rosuvastatin was significant (5.039; 95% confidence interval [CI], 1.091–23.268). With the other statins, only the statin index for pravastatin tended to decrease the risk of GI bleeding (1.280; 95% CI, 1.038–1.578). In these regression models, the order of warfarin and statin prescription neither affected the risk of GI bleeding with each statin nor had an independent effect on the risk of GI bleeding.

**Table 3:** Cox proportional hazards regression analysis of gastrointestinal bleeding for each statin group during the observation period.

Variables	Pravastatin group vs the others (n = 574)		Simvastatin group vs the others (n = 634)		Atorvastatin group vs the others (n = 1,432)		Rosuvastatin group vs the others (n = 732)	
	HR (95% CI)	p-value	HR (95% CI)	p-value	HR (95% CI)	p-value	HR (95% CI)	p-value
**Age**	1.045 (0.979–1.115)	0.189	1.003 (0.943–1.067)	0.920	1.041 (0.996–1.088)	0.076	1.072 (0.994–1.157)	0.072
**Male sex**	1.090 (0.329–3.605)	0.889	1.398 (0.400–4.891)	0.600	1.503 (0.673–3.356)	0.321	0.839 (0.259–2.716)	0.769
**Charlson comorbidity index**	1.047 (0.710–1.542)	0.818	1.126 (0.764–1.660)	0.550	1.295 (1.047–1.601)	0.017	1.143 (0.758–1.724)	0.524
**Nonsteroidal anti-inflammatory drug exposure**	0.713 (0.087–5.875)	0.753	1.090 (0.130–9.147)	0.937	0.359 (0.048–2.681)	0.318	1.266 (0.155–0.363)	0.826
**Steroid exposure**	6.211 (0.661–58.342)	0.110	2.108 (0.260–17.071)	0.485	2.164 (0.285–16.422)	0.456	1.882 (0.220–16.100)	0.564
**Antiplatelet exposure**	1.804 (0.565–5.757)	0.319	0.926 (0.273–3.142)	0.902	1.505 (0.683–3.316)	0.311	0.273 (0.079–0.941)	0.040
**Proportion of time in the therapeutic range of warfarin**	1.161 (0.200–6.731)	0.868	1.962 (0.340–11.314)	0.451	1.262 (0.395–4.038)	0.695	0.723 (0.116–4.508)	0.729
**Warfarin prescription prior to statin prescription**	1.253 (0.393–3.997)	0.703	0.849 (0.240–3.003)	0.799	0.652 (0.273–1.559)	0.336	0.431 (0.108–1.727)	0.235
**Statin index**	0.262 (0.068–1.004)	0.051	0.838 (0.255–2.757)	0.771	1.238 (0.580–2.641)	0.581	5.394 (1.168–24.916)	0.031

HR, hazard ratio; CI, confidence interval

## Discussion

In the current study that aimed to determine the risk of GI bleeding with the combined use of a statin and warfarin, the risk of GI bleeding differed based on the statin. There was an increased risk of GI bleeding with rosuvastatin, while pravastatin tended to reduce the risk.

Statins represent some of the most widely prescribed medications owing to their pleiotropic effects and clinical efficacy [25], and treatment with statins has been associated with a reduced incidence of coronary artery disease and stroke [26, 27]. Warfarin is used to prevent embolic events in the vascular system [1–4]. The concomitant use of warfarin and statins is indicated for ischemic congestive heart failure, with prescription of a statin for coronary artery occlusive disease and warfarin for prevention of a stroke from a cardioembolism [28, 29]; peripheral artery disease [30, 31]; and previously used method of prevention for symptomatic intracranial atherosclerotic disease [32, 33]. Rosuvastatin was associated with an increased risk of GI bleeding compared with propensity score-matched controls in the present study. To the best of our knowledge, there have been no reports of a relationship between rosuvastatin and GI bleeding; however, we suspect that low cholesterol levels might underlie the relationship observed in the present study. Among the 4 statins in the present study, the incidence of GI bleeding was the highest in patients taking rosuvastatin, followed by atorvastatin, simvastatin, and pravastatin; however, the mean cholesterol level during the observation period was the lowest in the rosuvastatin group, followed by the atorvastatin, simvastatin, and pravastatin groups. These findings are similar with those of a previous study in which digestive mortality increased as the total cholesterol level decreased [34]. In this same study, low cholesterol levels in the cerebral arterioles were also related with intracerebral hemorrhages [34], and the occurrence of intracerebral hemorrhages reportedly increased with a decrease in total cholesterol levels [35]. Cholesterol is a key component of cellular membranes, and low cholesterol levels might weaken the cellular structure [36] of GI mucosal cells, resulting in a higher incidence of GI bleeding.

On the other hand, because the incidence of GI bleeding began to differ in the early observation period, a potential drug interaction cannot be ignored, despite the controversy that exists regarding a drug interaction between rosuvastatin and warfarin [37–39]. In young healthy subjects, a drug interaction is unlikely [39]. Nonetheless, in a large randomized study of rosuvastatin, INR dramatically increased after administration of rosuvastatin in a case who had continuously taken warfarin [37]. The exact mechanism of drug interactions between warfarin and rosuvastatin is unclear because metabolism via the cytochrome P450 enzyme, 2C9, is known to be minor [40]. Because the antithrombotic effect of warfarin might increase with administration of rosuvastatin [11], close monitoring of INR is recommended. A previous review article indicated that rosuvastatin might have a greater effect on INR than other statins [40], which is supported by the present results.

Interactions between warfarin and many drugs and some foods [41], which occur via the cytochrome P450 system, are concerning [42]; these interactions can reduce the preventive effects of warfarin, potentially resulting in a thromboembolic event and increasing the incidence of bleeding [43–45]. Of the potential life-threatening bleeding events, GI bleeding is the most common [46]. The interaction between statins that undergo P450-related metabolism and warfarin can reportedly increase the risk of GI bleeding [17]; this has been reported for atorvastatin, which is an inhibitor of CYP3A4 [17]. However, this result is controversial; compared with the other statins in the current study, atorvastatin was not associated with an increased incidence of GI bleeding.

The risk of GI bleeding was the lowest in the present study with pravastatin, which supports the findings of a previous study [17]. The first possible explanation is the effect of lower cholesterol levels, as already described. Of the 4 statins, pravastatin decreased the mean cholesterol levels the least during the observational period. Second, it is not likely that a drug interaction influenced the incidence of GI bleeding. A relationship between the cytochrome P450 system and pravastatin is not supported by the evidence [47].

Regarding the use of EHR data for analyses, secondary use or re-use of clinical data has been increasingly adopted with the growing availability of EHR. However, elaborate processing is required and is a major barrier to extracting clinical knowledge from the EHR because the primary intended purpose of EHRs is not clinical research. Detailed operational definitions, such as that for continuous administration in the present study, for fitting EHR data into an epidemiologic study design is a key process. In addition, selection of the proper epidemiologic design for both the aim of the study and the given dataset can affect the final results. In this study, we used within-class comparisons because they are less prone to the selection bias inherent in observational studies using EHR data than are comparisons of different medication classes. The use of an EHR might enable more efficient retrospective research such as outcomes research, comparative effectiveness research, and drug surveillance.

There are several limitations in the current study. First, this study was retrospectively performed in one institute. Therefore, the results cannot be easily generalized because warfarin control might differ among institutes and clinical settings. In our hospital, anticoagulant clinics have not been much used for control purposes. The proportion of time within the target INR range was relatively low compared with that in previous randomized control trials for anticoagulation. Second, the purpose of our study was to evaluate the effect of statins in patients taking warfarin; therefore, there might be some statistical error for the general risk factors for GI bleeding. However, we tried to avoid this by including patients who had been using warfarin for a long period. Third, we used hospital data over a period of 17 years. During that time, new drugs were developed, guidelines for statin or warfarin indications were updated, and GI prophylactic drugs were introduced, which could have affected our results. Therefore, we tried to adjust for a number of confounding factors in the analyses. Last, the Cox proportional hazard regression analyses were not adjusted for the use of GI prophylaxis drugs due to the multicollinearity between the use of these drugs and peptic ulcer disease, which is an element in the CCI calculation. If highly correlated variables are included in the regression model, the estimated coefficient could be biased. Therefore, we excluded the use of GI prophylaxis drugs because we used the CCI in the Cox model.

## Conclusions

Rosuvastatin increased the incidence of GI bleeding in patients taking warfarin over a lengthy observation period. In contrast, the relationship between pravastatin and GI bleeding was the weakest in patients taking warfarin. A prospective observational study is necessary to confirm our results.

## Supporting Information

S1 File STROBE statement. (DOCX)
