# Supplementary material for: Comparison of the Risk of Gastrointestinal Bleeding among Different Statin Exposures with Concomitant Administration of Warfarin: Electronic Health Record-Based Retrospective Cohort Study
Source: PLoS One. 2016 Jul 7;11(7):e0158130. doi: 10.1371/journal.pone.0158130 (PMC4936673; doi:10.1371/journal.pone.0158130)
Supplement: S1 File — (DOCX) [file pone.0158130.s001.docx]

STROBE Statement—checklist of items that should be included in reports of observational studies

|  | Item No. | Recommendation | Page  No. | Relevant text from manuscript |
| --- | --- | --- | --- | --- |
| **Title and abstract** | 1 | (*a*) Indicate the study’s design with a commonly used term in the title or the abstract | 1-2 | electronic health record-based retrospective cohort study |
|  |  | (*b*) Provide in the abstract an informative and balanced summary of what was done and what was found | 2-3 | There was a relatively high risk of gastrointestinal bleeding with rosuvastatin when administered concomitantly with warfarin. |
| Introduction | | | |  |
| Background/rationale | 2 | Explain the scientific background and rationale for the investigation being reported | 4-5 | However, there is controversy regarding this side effect, because other evidence suggests that statins might decrease GI bleeding in patients treated with warfarin. |
| Objectives | 3 | State specific objectives, including any prespecified hypotheses | 4-5 | Using EHR data, this study aimed to compare the risk of GI bleeding among four different statins (simvastatin, atorvastatin, pravastatin, and rosuvastatin) when co-administered with warfarin for at least a 30-day period and adjusting for other concomitant medications and baseline characteristics. |
| Methods | | | |  |
| Study design | 4 | Present key elements of study design early in the paper | 5 | This is a single-hospital retrospective cohort study. |
| Setting | 5 | Describe the setting, locations, and relevant dates, including periods of recruitment, exposure, follow-up, and data collection | 5-7 | Data source  Patient selection and cohort definition |
| Participants | 6 | (*a*) *Cohort study*—Give the eligibility criteria, and the sources and methods of selection of participants. Describe methods of follow-up  *Case-control study*—Give the eligibility criteria, and the sources and methods of case ascertainment and control selection. Give the rationale for the choice of cases and controls  *Cross-sectional study*—Give the eligibility criteria, and the sources and methods of selection of participants | 5-7 | Patient selection and cohort definition |
|  |  | (*b*) *Cohort study*—For matched studies, give matching criteria and number of exposed and unexposed  *Case-control study*—For matched studies, give matching criteria and the number of controls per case | 7-8 | Statistical analysis |
| Variables | 7 | Clearly define all outcomes, exposures, predictors, potential confounders, and effect modifiers. Give diagnostic criteria, if applicable | 5-8 | Patient selection and cohort definition  Statistical analysis |
| Data sources/ measurement | 8* | For each variable of interest, give sources of data and details of methods of assessment (measurement). Describe comparability of assessment methods if there is more than one group | 5-8 | Data source |
| Bias | 9 | Describe any efforts to address potential sources of bias | 7-8 | Statistical analysis |
| Study size | 10 | Explain how the study size was arrived at | 5 | Data source |

Continued on next page

| Quantitative variables | 11 | Explain how quantitative variables were handled in the analyses. If applicable, describe which groupings were chosen and why | 5-8 | Patient selection and cohort definition |
| --- | --- | --- | --- | --- |
| Statistical methods | 12 | (*a*) Describe all statistical methods, including those used to control for confounding | 7-8 | Statistical analysis |
|  |  | (*b*) Describe any methods used to examine subgroups and interactions |  | None |
|  |  | (*c*) Explain how missing data were addressed | 7-8 | Statistical analysis |
|  |  | (*d*) *Cohort study*—If applicable, explain how loss to follow-up was addressed  *Case-control study*—If applicable, explain how matching of cases and controls was addressed  *Cross-sectional study*—If applicable, describe analytical methods taking account of sampling strategy | 5-8 | Patient selection and cohort definition |
|  |  | (*e*) Describe any sensitivity analyses |  | None |
| Results | | | | |
| Participants | 13* | (a) Report numbers of individuals at each stage of study—eg numbers potentially eligible, examined for eligibility, confirmed eligible, included in the study, completing follow-up, and analysed | 8-12 | Characteristics of the overall population |
|  |  | (b) Give reasons for non-participation at each stage | 6 | Definition of continuous administration, co-administration, and observation period for statins and warfarin. |
|  |  | (c) Consider use of a flow diagram | 6 | Overview of the study design to compare the gastrointestinal bleeding risk between 1 of 4 statins concomitantly administered with warfarin. |
| Descriptive data | 14* | (a) Give characteristics of study participants (eg demographic, clinical, social) and information on exposures and potential confounders | 8-12 | Characteristics of the overall population |
|  |  | (b) Indicate number of participants with missing data for each variable of interest |  | None |
|  |  | (c) *Cohort study*—Summarise follow-up time (eg, average and total amount) | 8-12 | Characteristics of the overall population |
| Outcome data | 15* | *Cohort study*—Report numbers of outcome events or summary measures over time | 13-14 | Incidence of gastrointestinal bleeding in patients administered both warfarin and a statin, based on the 3 steps of identification. |
|  |  | *Case-control study—*Report numbers in each exposure category, or summary measures of exposure |  |  |
|  |  | *Cross-sectional study—*Report numbers of outcome events or summary measures |  |  |
| Main results | 16 | (*a*) Give unadjusted estimates and, if applicable, confounder-adjusted estimates and their precision (eg, 95% confidence interval). Make clear which confounders were adjusted for and why they were included | 13-16 | Cox proportional hazards regression analysis of gastrointestinal bleeding for each statin group during the observation period. |
|  |  | (*b*) Report category boundaries when continuous variables were categorized | 13-16 | Cox proportional hazards regression analysis of gastrointestinal bleeding for each statin group during the observation period. |
|  |  | (*c*) If relevant, consider translating estimates of relative risk into absolute risk for a meaningful time period | 13-16 | Cox proportional hazards regression analysis of gastrointestinal bleeding for each statin group during the observation period. |

Continued on next page

| Other analyses | 17 | Report other analyses done—eg analyses of subgroups and interactions, and sensitivity analyses |  | None |
| --- | --- | --- | --- | --- |
| Discussion | | | | |
| Key results | 18 | Summarise key results with reference to study objectives | 17 | In the current study that aimed to determine the risk of GI bleeding with the combined use of a statin and warfarin, the risk of GI bleeding differed based on the statin. |
| Limitations | 19 | Discuss limitations of the study, taking into account sources of potential bias or imprecision. Discuss both direction and magnitude of any potential bias | 19-20 | There are several limitations in the current study. |
| Interpretation | 20 | Give a cautious overall interpretation of results considering objectives, limitations, multiplicity of analyses, results from similar studies, and other relevant evidence | 17-20 | Discussion |
| Generalisability | 21 | Discuss the generalisability (external validity) of the study results | 17-20 | Discussion |
| Other information | |  | | |
| Funding | 22 | Give the source of funding and the role of the funders for the present study and, if applicable, for the original study on which the present article is based | In the online metadata |  |

*Give information separately for cases and controls in case-control studies and, if applicable, for exposed and unexposed groups in cohort and cross-sectional studies.

**Note:** An Explanation and Elaboration article discusses each checklist item and gives methodological background and published examples of transparent reporting. The STROBE checklist is best used in conjunction with this article (freely available on the Web sites of PLoS Medicine at http://www.plosmedicine.org/, Annals of Internal Medicine at http://www.annals.org/, and Epidemiology at http://www.epidem.com/). Information on the STROBE Initiative is available at www.strobe-statement.org.
